# Calidad de la mamografía y tamizaje del cáncer de mama en Argentina

**DOI:** 10.26633/RPSP.2019.63

**Published:** 2019-07-31

**Authors:** Susana Blanco, Daniel Andisco, Pablo Jiménez, Silvana Luciani

**Affiliations:** 1 Instituto Nacional del Cáncer Instituto Nacional del Cáncer Secretaría de Salud de la Nación Buenos Aires Argentina Instituto Nacional del Cáncer, Secretaría de Salud de la Nación, Buenos Aires, Argentina.; 2 Organización Panamericana de la Salud Organización Panamericana de la Salud Washington, D.C. Estados Unidos de América. Organización Panamericana de la Salud, Washington, D.C., Estados Unidos de América.

**Keywords:** Mamografía, cáncer de mama, garantía de la calidad de atención de salud, acreditación, Argentina, Mammography, breast neoplasms, quality assurance, health care, accreditation, Argentina

## Abstract

Se presentan los resultados del Plan de calidad en mamografía del Programa Nacional del Cáncer de Mama del Instituto Nacional del Cáncer de la República Argentina, enfocado en mejorar la calidad de la mamografía en el sector público de salud e implementar el tamizaje de cáncer de mama por este método diagnóstico. El Plan se comenzó a ejecutar en 2011 con base en la premisa de que una mamografía de mala calidad impide el diagnóstico temprano del cáncer de mama. En ese momento, en Argentina existía poca conciencia sobre la importancia de los controles de calidad, y los continuos cambios en los niveles jerárquicos del sistema de salud tendían a obstaculizar el desarrollo organizado del programa. El Plan consistió en la revisión y el control de calidad de las instalaciones de mamografía, la capacitación de recursos humanos técnicos y médicos y la puesta en marcha de un sistema de acreditación de los servicios de mamografía por parte del Instituto Nacional del Cáncer. La percepción de la importancia de la calidad en el personal involucrado ha mejorado y se obtuvo un panorama general del estado de la mamografía a nivel nacional en cuanto a la calidad, la tecnología, la cantidad de equipos y las posibilidades de implementación del tamizaje. Se capacitó al personal técnico y médico mediante el uso de diferentes metodologías y se impulsó la unificación de la lectura mamográfica en las provincias intervinientes.

## Abstract (en)

This paper presents results from the mammography quality plan of the National Breast Cancer Program at Argentina’s National Cancer Institute, which focused on improving mammography quality in the public health-care sector and on using mammography to implement breast cancer screening. Plan implementation started in 2011, based on the premise that poor quality mammography impedes early diagnosis of breast cancer. At the time, there was little awareness in Argentina of the importance of quality control, and constant changes in lines of authority in the health system tended to hinder organized implementation of the program. The plan consisted of quality review and control of mammography facilities, training of technical and medical human resources, and implementation of an accreditation system for mammography services by the National Cancer Institute. Perception by involved personnel of the importance of quality improved; an overview was obtained of the state of mammography at the national level regarding quality, technology, quantity of equipment, and opportunities for implementation of screening; technical and medical personnel were trained using different methods; and standardization of mammography reading was promoted in the participating provinces.

## RESUMO

São apresentados os resultados do Plano de qualidade em mamografia do Programa Nacional do Câncer de Mama do Instituto Nacional do Câncer da República Argentina, com o objetivo de melhorar a qualidade da mamografia na rede pública de saúde e implementar a prevenção do câncer de mama por este método diagnóstico. O Plano foi implementado em 2011 baseado na premissa de que a mamografia de má qualidade impede o diagnóstico precoce do câncer de mama. Havia, naquele momento na Argentina, pouca conscientização sobre a importância do controle de qualidade e as constantes mudanças na hierarquia do sistema de saúde eram um obstáculo ao desenvolvimento organizado do programa. O Plano compreendeu a avaliação e o controle de qualidade das instalações para mamografia, a capacitação do pessoal médico e técnico e a implantação de um sistema de credenciamento pelo Instituto Nacional do Câncer dos serviços de mamografia. Houve melhora na percepção da importância da qualidade entre o pessoal envolvido e se obteve um panorama geral da situação da mamografia ao nível nacional quanto à qualidade, recursos tecnológicos, volume de equipamentos e possibilidades de implementação do programa de prevenção. Foi realizada a capacitação do pessoal médico e técnico com o uso de diferentes metodologias e incentivada a uniformização da leitura mamográfica nas províncias consideradas.

La mortalidad por cáncer de mama muestra una amplia variabilidad entre diferentes regiones y se estima que, en América Latina, aproximadamente 52 550 mujeres mueren cada año por esta enfermedad (1). Argentina tiene la segunda tasa de mortalidad en la Región –después de Uruguay– con una tasa de 18 por cada 100 000 en 2018, lo que representa 6 380 muertes (1).

Es posible controlar el cáncer de mama con un diagnóstico temprano y un tratamiento adecuado. En países industrializados, donde se han implementado programas de tamizaje, se ha demostrado un descenso de 20% en la mortalidad durante 11 años de seguimiento (2).

El control del cáncer de mama en Argentina, considerado un país no industrializado con recursos medianos, se debe abordar a partir de programas de información, educación y consejería que aumentan el conocimiento de las mujeres sobre los factores de riesgo y los signos y síntomas que requieren atención médica inmediata. Estos programas deben ser complementados con un tamizaje organizado para detectar el cáncer en los estadios iniciales. Todo ello implica disponer de un sistema de salud sólido con una amplia gama de recursos financieros, humanos y educativos y con una llegada eficiente a la población.

La Resolución CSP28.R15 de la Conferencia Sanitaria Panamericana (Washington, D.C., Estados Unidos de América, 2012), *Protección radiológica y seguridad de las fuentes de radiación: normas básicas internacionales de seguridad* (3), incluye requisitos específicos para la protección de los pacientes. La exposición a la radiación por motivos médicos requiere programas integrales de garantía de calidad y consideraciones especiales para justificar los programas de tamizaje.

Un estudio multicéntrico realizado por la Organización Panamericana de la Salud (OPS) en Argentina, Bolivia, Colombia, Cuba y México demostró que existe una relación directa entre la calidad de las imágenes radiográficas y la certeza en la interpretación radiológica. En las 366 imágenes de mamografía estudiadas se comprobó concordancia entre la interpretación radiológica efectuada por un panel de expertos y la del médico local en 75% de los casos (4).

Varios países de América Latina han seguido diferentes caminos y se encuentran en distintas etapas de implementación de políticas sobre el control del cáncer de mama. Mientras que Brasil y México han establecido políticas nacionales integrales, Argentina, Colombia y Venezuela han logrado avances importantes pero aún no han logrado coordinar políticas nacionales (5).

Con el objetivo de mejorar los sistemas existentes en la detección temprana del cáncer de mama, en Argentina se inició un trabajo de concientización sobre la calidad, capacitación de recursos humanos y relevamiento del estado de los equipos instalados financiado por el Programa Nacional de Control de Cáncer de Mama (PNCM) del Instituto Nacional del Cáncer (INC). Este depende de la Secretaría de Salud de la Nación y sigue las recomendaciones de la OPS de ofrecer la mamografía cada dos años a las mujeres a partir de los 50 años y hasta los 69 años.

En 2010, se publicaron los resultados del relevamiento de la situación del cáncer de mama en la Argentina (6). Si bien el análisis se centró en las provincias con alta incidencia de la enfermedad, el relevamiento fue general y abarcó todo el país. El informe reportó una heterogeneidad marcada de accesibilidad a los mamógrafos en las diferentes provincias del país. Aunque las 24 provincias argentinas contaban con mamógrafos en el sector público, la cantidad de equipos en funcionamiento variaba de una a otra. En algunos casos, la cantidad de equipos era suficiente para cubrir las necesidades de un tamizaje masivo, mientras que en otros solo permitía asegurar una cobertura cercana a la mitad de la población objetivo (6). En todas las provincias, los mamógrafos carecían de mantenimiento preventivo y no se llevaban a cabo controles de calidad. Ninguna provincia tenía registro de controles de calidad sistemáticos. Por otra parte, la mayoría de los equipos estaban subutilizados y funcionaban durante pocas horas al día. En las zonas alejadas de la capital provincial y de difícil acceso había gran demora en la obtención de insumos y en el mantenimiento y reparación de los equipos. También se detectó un retraso de hasta 150 días desde la realización del estudio mamográfico hasta la implementación del tratamiento (6).

A fin de reducir la mortalidad y la morbilidad por cáncer de mama, el PNCM comenzó una intervención que abarcó todos los elementos que interferían con la detección temprana de la enfermedad en el sector público de salud. Se diseñó un plan de calidad dentro del PNCM para la monitorización continuada de los equipos y servicios de mamografía y para capacitar a los recursos humanos. Se consideró fundamental la capacitación de los médicos radiólogos en la lectura y el informe de la mamografía de tamizaje, así como la uniformidad en el modo de reportar los resultados de las mamografías.

El objetivo de este artículo es presentar los resultados del Plan de calidad en mamografía del Programa Nacional de Control de Cáncer de Mama del Instituto Nacional del Cáncer de la República Argentina. Este consistió en la revisión y el control de calidad de instalaciones de mamografía, la capacitación de recursos humanos técnicos y médicos y la puesta en marcha de un sistema de acreditación de los servicios de mamografía por parte del INC.

## MÉTODOS

El Plan de calidad comenzó en 2011 con visitas del equipo de física médica a las provincias adheridas al PNCM. Esta adhesión era voluntaria y solo participaron ocho de las 24 provincias del país. Las visitas confirmaron varios problemas, inclusive: 1) variabilidad de las tecnologías disponibles –tanto analógicas como de radiología computarizada (RC) y de radiología directa (RD)– según el hospital y la provincia; 2) defectos graves en la calidad de imagen en los equipos analógicos y de RC; 3) variabilidad en las dosis de radiación emitidas en cada estudio y en cada centro de atención pública del país, algunas de las cuales estaban fuera del nivel máximo recomendado (< 2 mGy); 4) desconocimiento por parte de los técnicos radiólogos de las características de los equipos de mamografía con los cuales trabajan y de las técnicas de posicionamiento de la mama; y 5) falta de uniformidad en la técnica de categorización de patologías mamarias (muchos médicos no utilizaban el sistema *Breast Imaging Reporting and Data System,* BI-RADS) (7).

El PNCM y el equipo del Plan de calidad establecieron las acciones prioritarias necesarias para alcanzar una infraestructura de calidad que permitiera implementar un programa de tamizaje organizado en algunas regiones del país. Estas acciones tuvieron en cuenta los recursos del INC y las diferentes características regionales, e incluyeron la concientización sobre la importancia de la calidad mamográfica, las visitas a los servicios de mamografía, la capacitación de los técnicos radiólogos, la implementación de un programa de doble lectura mamográfica y la implementación de un programa de garantía de calidad en los servicios de mamografía públicos.

### Importancia de la calidad mamográfica

El sector público de salud en Argentina no posee los recursos suficientes para implementar sistemas y controles de la calidad mamográfica para el diagnóstico temprano del cáncer de mama en todos los servicios. Con el objetivo de concientizar sobre la importancia de este aspecto del diagnóstico por mamografía, se organizaron talleres presenciales dirigidos a médicos, técnicos radiólogos y autoridades de centros de diagnóstico y de salud, en los que se presentó el Plan de calidad y se enfatizó la relación entre la calidad de las imágenes mamográficas y la necesidad de realizar controles de calidad periódicos de los equipos.

También se destacó la importancia de adherir al registro de pacientes del INC, llamado Sistema de Tamizaje (SITAM), que reúne los datos de las mujeres desde el inicio de los cuidados de su salud mamaria (mamografía) y permite el seguimiento y la detección temprana de demoras en el diagnóstico o el tratamiento (ecografía, histopatología, inmunohistoquímica, cirugía, radioterapia y quimioterapia).

### Visitas a los servicios de mamografía

Entre 2012 y 2015 se visitaron los centros de salud adheridos al Plan de calidad. En estas visitas se realizaron controles de calidad de los mamógrafos para establecer su estado funcional y se tomaron imágenes de muestra para conocer la forma de trabajo de los técnicos radiólogos. Es importante mencionar que, con excepción de los grandes hospitales de las capitales provinciales, en muchas localidades no hay médicos radiólogos en los servicios en forma permanente, y son los técnicos radiólogos quienes determinan la necesidad de repetir o no una exposición.

Al momento de iniciar las visitas, se desconocía el número de mamógrafos instalados. Dos físicos médicos del PNCM visitaron y controlaron 138 equipos entre los años 2012 y 2017; algunos servicios fueron visitados en más de una oportunidad debido a cambios o reparaciones.

El control de calidad y la medición de la dosis glandular media se efectuaron según las recomendaciones de las guías para el control de calidad de equipos analógicos y digitales publicados por el Organismo Internacional de Energía Atómica y el manual de tamizaje para América Latina y el Caribe de la OPS (8-10). Este control primario se realizó por única vez y en la visita se brindó capacitación a los técnicos a fin de que efectuaran controles periódicos que no requieren un equipamiento específico.

Se elaboró un informe de cada equipo inspeccionado. Los informes se elevaron a las autoridades del servicio de mamografía del hospital y a las autoridades de salud locales; en ellos se recomendaba la compra de equipamiento para controles de calidad y la capacitación de físicos médicos.

### Capacitación de técnicos radiólogos

Durante las visitas a los servicios, se detectaron deficiencias en la capacitación de los técnicos radiólogos mediante la obtención de muestras de las imágenes (20 en cada centro, correspondientes al mes anterior a la visita).

Los equipos no eran utilizados en su máximo potencial, no había manuales de funcionamiento y, cuando no funcionaba el control automático de exposición del mamógrafo, empeoraba la calidad de las placas y aumentaban las repeticiones que, además, no eran registradas.

### Implementación de un programa de doble lectura mamográfica

El número elevado de estudios informados como BI-RADS 0 detectado a través del SITAM, junto con la falta de calidad de las imágenes mamográficas, fueron indicadores de dificultades en el diagnóstico y el tratamiento futuro.

Durante las visitas a los servicios se detectó que en las localidades en las que no había médicos radiólogos no se utilizaba el sistema BI-RADS, y resultó evidente la necesidad de unificar la forma de lectura según esta categorización. Se organizaron ejercicios de doble lectura con la colaboración de dos médicos radiólogos del Plan de calidad del PNCM para formar radiólogos referentes en las provincias que integran el quintil más elevado de mortalidad por cáncer de mama (Mendoza, San Luis y Misiones con una tasa ajustada por edad de 10,79-20,97/ 100 000 mujeres, frente al quintil más bajo en Jujuy, La Rioja, Santiago del Estero, Santa Cruz y Tierra del Fuego).

El sistema consistió en la visita de un médico radiólogo del INC a las provincias que adhirieron al ejercicio para realizar la doble lectura en un conjunto de 30 estudios categorizados desde BI-RADS 0 hasta BI-RADS 5 por nuestros dos expertos y tres médicos consultantes. El conjunto de mamografías incluía todas aquellas imágenes en las que los dos expertos y los tres consultantes habían coincidido. Como indicador de este ejercicio, se tomó el porcentaje de acuerdo entre el radiólogo del INC y el médico local en el 80% de las lecturas.

El programa de visitas se interrumpió en 2015 por razones presupuestarias y para compensarlo se implementó un curso virtual de actualización sobre el sistema de categorización BI-RADS. Este curso es gratuito y de libre acceso y se dicta dos veces al año (11).

### Implementación de un programa de garantía de calidad

La experiencia adquirida hasta 2014 demostró que la calidad de la mamografía constituye un tema integral que abarca desde la asignación de turnos hasta la entrega de los resultados en tiempo y forma.

En la búsqueda de una solución integral para definir los centros de tamizaje, los físicos médicos del PC redactaron *Recomendaciones para el correcto funcionamiento de un servicio de mamografía* (12), que se pusieron en práctica hacia finales de 2015 bajo la supervisión del PC y la auditoría del INC. En este documento, se incluyeron recomendaciones de procedimientos en el servicio y un cronograma de controles de calidad.

Antes de cada auditoría, en la que se somete al equipo de mamografía a un exhaustivo control de calidad, el PNCM verifica que las instalaciones sean las apropiadas para un servicio de mamografía y estén habilitadas por las autoridades locales. Luego, el personal debe redactar un manual de calidad y los procedimientos del servicio, los controles de calidad, los procedimientos de prácticas y la atribución de responsabilidades, entre otros aspectos.

El funcionamiento correcto del equipo (verificado por el control de calidad que realizan los auditores, la documentación y un acuerdo de 80% de los informes mamográficos con los efectuados por médicos consultores expertos del Plan de calidad) es una condición excluyente para la acreditación del servicio de mamografía de la institución.

## RESULTADOS Y DISCUSIÓN

### Importancia de la calidad mamográfica

El INC convocó a jefes de servicios de mamografía, técnicos radiólogos y médicos informantes de estudios a los talleres en una provincia central de cada región a lo largo de dos días. Durante estas reuniones se impartieron clases de temáticas afines: tipos de tecnologías de mamógrafos, radiobiología, protección radiológica, técnica mamográfica, calidad de imagen en mamografía y clasificación BIRAD.

### Visitas a los servicios de mamografía

Según el censo realizado por el INC en 2018 (13), existen 377 mamógrafos en el sistema público. De estos, 70% son analógicos, 23% son digitalizados y 7% son digitales. La cantidad de equipos visitados representa 45% de los mamógrafos instalados en 18 de las 24 provincias (Buenos Aires, Catamarca, Chaco, Chubut, Ciudad Autónoma de Buenos Aires, Corrientes, Entre Ríos, Jujuy, La Pampa, Mendoza, Misiones, Neuquén, Río Negro, San Juan, San Luis, Santa Fe, Santiago del Estero y Tucumán) y la muestra mantuvo un porcentaje similar de tipos de tecnología instalada (68% tenía tecnología analógica, el 22% eran RC y el 10% eran RD).

Estos equipos han pasado por estrictos controles de calidad al menos una vez. Se han gestionado las correcciones a los problemas encontrados durante reuniones con las autoridades locales o de las instituciones visitadas.

### Capacitación de técnicos radiólogos

Durante 2011, se realizaron cinco talleres en las regiones noreste, noroeste, sur, centro y este de Argentina.

Desde sus comienzos, el INC cuenta con un programa de becas para capacitar técnicos y médicos en la Ciudad Autónoma de Buenos Aires (se formaron 21 médicos radiólogos y 20 técnicos radiólogos). Sin embargo, ese programa –que se desarrolla en centros de excelencia– no permitía la reinserción adecuada del profesional en su antiguo lugar de trabajo, dada la diferencia de rutinas y equipamientos. Se decidió entonces diseñar un sistema de capacitación con la presencia de una técnica radióloga perteneciente al Plan de calidad, que brindara capacitación en el lugar de trabajo. De esta manera, las técnicas en mamografía locales llegaban a conocer sus equipos y se perfeccionaban en el posicionamiento de la mama durante el estudio (los errores en este último son responsables de la mayoría de las repeticiones). Para apoyar esta capacitación y llegar a los lugares más apartados del país se desarrollaron dos cursos virtuales de libre acceso sobre la física de la mamografía y el posicionamiento de la mama (14, 15). También se elaboró material de apoyo de libre acceso sobre tecnología y controles básicos de calidad dirigido a técnicos radiólogos (16, 17).

Mediante un sistema de capacitación en el lugar de trabajo se formaron 82 nuevos técnicos radiólogos. Se desconoce el número exacto total de técnicos radiólogos, ya que no hay registros del personal técnico activo y tampoco lo hay de médicos informantes. Durante 2019 el PNCM está realizando un censo de recursos humanos dedicados a mamografía para establecer indicadores confiables.

### Implementación de un programa de doble lectura mamográfica

En el programa de doble lectura mamográfica participaron 23 médicos radiólogos, que se desempeñan en hospitales públicos de referencia en las provincias de La Pampa, Mendoza, Misiones, Neuquén, San Juan, San Luis y Río Negro.

A los médicos locales se les envió previamente un grupo de placas armado por los especialistas del INC. Más tarde, estos mismos especialistas viajaron a las instituciones provinciales para hacer la doble lectura.

### Implementación de un programa de garantía de calidad

En la actualidad, hay acreditados ocho servicios de mamografía correspondientes a los respectivos hospitales provinciales: uno en Mendoza, dos en Río Negro, uno en Neuquén, uno en Misiones, dos en Santa Fe y uno en Chubut. Durante 2019 se continuará el proceso en los hospitales que cumplan las condiciones solicitadas.

A partir de la implementación de este plan de calidad, se han observado cambios en los servicios de mamografía del sistema público de salud en Argentina. La medida de este cambio es la cantidad de provincias adheridas (en las que al menos uno de sus hospitales es acreditado según las normas de calidad del INC). Desde el comienzo del programa, la adhesión ha pasado de 8 a 18 provincias y las provincias han aumentado los requerimientos al INC de visitas y controles. Asimismo, existe una mayor conciencia de la relación entre la calidad mamográfica y el diagnóstico temprano.

Los cambios continuos en los programas de la Secretaría de Salud han complicado en algunos momentos el cumplimiento de todos los objetivos propuestos por el plan de calidad por las naturales consecuencias del cambio de autoridades y durante estos seis años el INC ha sufrido altibajos en el presupuesto asignado.

La falta de físicos médicos especializados en radiodiagnóstico y de equipamiento para realizar controles de calidad son una barrera importante en el logro de los objetivos. Solo las provincias de Córdoba, San Luis, Santa Fe, San Luis y Mendoza cuentan con objetos de prueba y equipos para controlar los mamógrafos.

El soporte a la actualización de contenidos ofrecido por los cursos virtuales ha sido recibido con beneplácito y el cupo de 120 postulantes por curso semestral se completa, en todos los casos, con participantes de todo el país.

Muchos centros mamográficos del interior del país trabajan solo un turno de cuatro o seis horas. En algunos casos, hay un solo médico en un centro único de lectura que se hace cargo de las mamografías de una provincia o región provincial. En general, el INC desalienta la utilización de unidades móviles de mamografía (“camiones mamográficos”) por la dificultad de su mantenimiento; sin embargo, estas se siguen utilizando. Es así que, a pesar de las recomendaciones, llegan mamografías de muy baja calidad de imagen a los centros de lectura (18).

Si bien las regiones con población nominalizada (población en la que se conoce si pertenece o no al grupo de edad objetivo [50-69 años] y es localizable por domicilio) están identificadas, los equipos técnicos y los mamógrafos aún no cumplen con los criterios mencionados en el manual de tamizaje de la OPS para implementar un ejercicio piloto organizado.

A nivel regional, solo 14 de 33 países en América Latina y el Caribe informan que poseen servicios de mamografía para el tamizaje y detección temprana de cáncer de mama, y solo tres países (Brasil, Cuba y Uruguay) reportaron que han alcanzado la cobertura necesaria (de 70% o más) de la población objetivo (19).

Uno de los mayores inconvenientes que se ha encontrado en Argentina es el alto porcentaje de equipos analógicos (70,2%). Debe promoverse un cambio de tecnología para discontinuar el proceso de revelado, que es perjudicial para el ambiente y no permite garantizar la necesaria calidad de las imágenes para tamizaje. La tecnología instalada presenta deficiencias para una identificación correcta de patologías en estadio temprano, ya que 40,7% de las imágenes obtenidas no presentaron los estándares de calidad apropiados para una mamografía de tamizaje. Desde 2018, el Plan de calidad trabaja en la fabricación de un objeto de prueba artesanal calibrado uno a uno y un sistema de dosimetría a distancia que comenzará a implementarse en 2019.

## DESAFÍOS FUTUROS Y CONCLUSIONES

El Plan de calidad que se desarrolló en Argentina permitió conocer las limitaciones para implementar programas de tamizaje organizados.

Un programa público de tamizaje orientado a reducir la mortalidad por cáncer de mama necesita recursos para la convocatoria de los pacientes y su traslado, el mantenimiento de los equipos y la disponibilidad de personal altamente capacitado. Los ministerios de salud tienen dificultades para incorporar controles obligatorios y periódicos de la calidad de los equipos, ya sea por falta de personal capacitado o por falta de equipamiento. El presupuesto de salud suele ser un eslabón débil en la cadena de prioridades.

Contar con un plan de calidad que asegure que las imágenes serán diagnósticas y luego garantizar a las mujeres con un diagnóstico patológico los cuidados necesarios hasta su recuperación implica una tarea constante.

La amplia extensión de Argentina implica costos y dificultades de traslado para el personal que efectúa los controles de calidad de los equipos. Asimismo, la falta de recursos obstaculiza la adquisición del equipamiento necesario para asegurar la calidad de funcionamiento de los mamógrafos. En la actualidad, en Argentina este equipamiento está disponible solo en las principales ciudades del país.

La definición de criterios sobre la obsolescencia de equipos mamográficos es una tarea que está en sus comienzos. Con estos criterios se espera impulsar cambios tecnológicos en las provincias para mejorar la calidad mamográfica y evitar el uso de sustancias químicas que afectan al medio ambiente. La OPS y el Organismo Internacional de Energía Atómica (OIEA) contribuyen con donaciones de equipos para estos fines, pero estos son insuficientes en países como Argentina, donde se concentran principalmente en su capital o en las ciudades más pobladas.

La falta de físicos médicos entrenados en radiodiagnóstico también es un problema presente en Argentina y en toda América Latina. Esto limita la calidad de las imágenes y, por ende, la implementación de programas de tamizaje.

Otra dificultad observada tanto en Argentina como en muchos otros países de la Región de las Américas es la deficiente capacitación de los técnicos radiólogos. Faltan programas actualizados de capacitación que incorporen los conceptos fundamentales de las nuevas tecnologías y establezcan una práctica extensa y supervisada de los profesionales técnicos. No todos los países poseen la cantidad necesaria de médicos radiólogos y, en las zonas más alejadas de los principales centros urbanos, es el técnico radiólogo quien toma las decisiones importantes.

Se requieren políticas sostenidas en el tiempo, controles de calidad y personal capacitado para establecer programas de tamizaje mamográfico que contribuyan a la reducción del cáncer de mama.

### Agradecimientos

SB agradece al Consejo Nacional de Investigaciones Científicas y Técnicas (CONICET) por su soporte y apoyo. DA y SB agradecen especialmente al Instituto Nacional del Cáncer (INC) y a María Viniegra por la concepción y el estímulo para realizar este trabajo.

### Financiación

Este estudio fue financiado por el Instituto Nacional del Cáncer de Argentina.

### Contribución de los autores

Los autores han contribuido en forma equitativa en la elaboración de este trabajo. SB y DA han realizado el trabajo de campo.

### Declaración

Las opiniones expresadas en este manuscrito son responsabilidad de los autores y no reflejan necesariamente los criterios ni la política de la *RPSP/PAJPH* y/o de la OPS.
